# Myocardial Infarction - Stress PRevention INTervention (MI-SPRINT) to reduce the incidence of posttraumatic stress after acute myocardial infarction through trauma-focused psychological counseling: study protocol for a randomized controlled trial

**DOI:** 10.1186/1745-6215-14-329

**Published:** 2013-10-11

**Authors:** Rebecca Meister, Mary Princip, Jean-Paul Schmid, Ulrich Schnyder, Jürgen Barth, Hansjörg Znoj, Claudia Herbert, Roland von Känel

**Affiliations:** 1 Department of General Internal Medicine, Division of Psychosomatic Medicine, Inselspital, Bern University Hospital, University of Bern, Bern, Switzerland; 2 Department of Clinical Research, University of Bern, Bern, Switzerland; 3 Institute of Psychology, Division of Clinical Psychology and Psychotherapy, University of Bern, Bern, Switzerland; 4 Department of Cardiology, Cardiovascular Prevention, Rehabilitation & Sports Medicine, Inselspital, Bern University Hospital, University of Bern, Bern, Switzerland; 5 Department of Psychiatry and Psychotherapy, University Hospital Zurich, Zurich, Switzerland; 6 Institute of Social and Preventive Medicine, University of Bern, Bern, Switzerland; 7 The Oxford Development Center Ltd, Oxfordshire, UK

**Keywords:** Anxiety disorder, Biomarkers, Cardiovascular disease, Counseling, Myocardial infarction, Posttraumatic stress disorder, Prevention, Psychological stress, Psychotherapy, Randomized controlled trial

## Abstract

**Background:**

Posttraumatic Stress Disorder (PTSD) may occur in patients after exposure to a life-threatening illness. About one out of six patients develop clinically relevant levels of PTSD symptoms after acute myocardial infarction (MI). Symptoms of PTSD are associated with impaired quality of life and increase the risk of recurrent cardiovascular events. The main hypothesis of the MI-SPRINT study is that trauma-focused psychological counseling is more effective than non-trauma focused counseling in preventing posttraumatic stress after acute MI.

**Methods/Design:**

The study is a single-center, randomized controlled psychological trial with two active intervention arms. The sample consists of 426 patients aged 18 years or older who are at 'high risk’ to develop clinically relevant posttraumatic stress symptoms. 'High risk’ patients are identified with three single-item questions with a numeric rating scale (0 to 10) asking about 'pain during MI’, 'fear of dying until admission’ and/or 'worrying and feeling helpless when being told about having MI’. Exclusion criteria are emergency heart surgery, severe comorbidities, current severe depression, disorientation, cognitive impairment and suicidal ideation. Patients will be randomly allocated to a single 45-minute counseling session targeting either specific MI-triggered traumatic reactions (that is, the *verum* intervention) or the general role of psychosocial stress in coronary heart disease (that is, the control intervention). The session will take place in the coronary care unit within 48 hours, by the bedside, after patients have reached stable circulatory conditions. Each patient will additionally receive an illustrated information booklet as study material. Sociodemographic factors, psychosocial and medical data, and cardiometabolic risk factors will be assessed during hospitalization. The primary outcome is the interviewer-rated posttraumatic stress level at three-month follow-up, which is hypothesized to be at least 20% lower in the *verum* group than in the control group using the *t*-test. Secondary outcomes are posttraumatic stress levels at 12-month follow-up, and psychosocial functioning and cardiometabolic risk factors at both follow-up assessments.

**Discussion:**

If the *verum* intervention proves to be effective, the study will be the first to show that a brief trauma-focused psychological intervention delivered within a somatic health care setting can reduce the incidence of posttraumatic stress in acute MI patients.

**Trial registration:**

ClinicalTrials.gov: NCT01781247

## Background

Posttraumatic Stress Disorder (PTSD) is a mental disorder that may occur after the experience of a traumatic event. According to the fourth edition of the *Diagnostic and Statistical Manual of Mental Disorders* (*DSM-IV*), PTSD is characterized by three distinct clusters of symptoms consisting of 1) re-experiencing of the traumatic event (for example, in thoughts or dreams), 2) avoidance of reminders of the event and emotional numbing and 3) hyperarousal (for example, irritability and sleep problems). These symptoms must last for at least one month and cause significant problems in important areas of daily life [1]. Besides man-made and disaster-related traumatic experiences (for example, combat exposure, motor vehicle accidents, hurricanes), life-threatening illnesses, such as myocardial infarction (MI), can also cause PTSD [2-6]. A meta-analysis on clinically significant PTSD symptoms after acute coronary syndromes (ACS) found a prevalence of 16% (95% CI, 13 to 20%) [7]; two-thirds of patients continue to suffer from PTSD two years after MI [8].

Several sociodemographic and psychosocial risk factors for the risk of MI-triggered PTSD have been identified, including young age [9] and increased distress perceived during MI [10]. While MI-related threat to life, helplessness and pain are predictive of PTSD, objective markers of myocardial damage such as cardiac enzyme levels and left ventricular ejection fraction are not predictive [9,11,12]. Less than optimal care processes like emergency department crowding might contribute to MI patients’ sense of life threat and decreased control, thereby increasing the risk of developing posttraumatic stress [13].

PTSD is associated with impaired quality of life, social functioning, and high economic burden to the society [14-16]. PTSD and posttraumatic stress along a continuum of severity are predictive of incident cardiovascular disease morbidity and mortality [17-20]. A meta-analysis on three prospective studies with a total of 609 ACS patients showed that PTSD symptoms also worsen cardiovascular prognosis with a two-fold (95% CI, 1.7 to 2.4) excess risk [7]. Moreover, this relationship is maintained in studies controlling for other prognostic risk factors, including traditional cardiovascular disease (CVD) risk factors, MI severity, and depressive symptoms [21-23].

PTSD might exert its 'cardiotoxic’ effect by several pathways and mechanisms. In patients with coronary heart disease (CHD), PTSD is associated with poor health behaviors, including low level of physical activity, smoking, and nonadherence with cardiac medications [24]. Commonly observed dysregulation of the hypothalamic-pituitary-adrenal axis and autonomic nervous dysfunction in PTSD might directly underlie a variety of physiological changes potentially increasing the atherothrombotic risk [25]. Accordingly, several cardiometabolic risk factors and markers are associated with PTSD: endothelial dysfunction [26], dyslipidemia [27], elevated resting blood pressure and heart rate [28], low-grade inflammation [29,30], and increased coagulation activity [31].

Because of its association with poor mental and cardiovascular health, the prevention of PTSD development after MI is of high clinical relevance. A systematic review and meta-analysis of randomized controlled trials of early psychological interventions to prevent symptoms of PTSD in individuals exposed to a specific traumatic event found a benefit for individuals with early symptoms of traumatic stress as opposed to those with no such symptoms and if the intervention was trauma-focused [32,33]. There was no evidence for a harmful effect of such early interventions as long as compulsory psychological debriefing of traumatized patients was avoided [33,34]. To improve adaptation to cardiac events, an approach that uses cognitive (re)structuring and an individual patient’s resources (for example, social support, distraction, structure, helping patients regaining control, building confidence in their future abilities to successfully cope with the MI, enhancing resilience) is warranted [35-37]. It follows from the above literature that to prevent PTSD symptoms after MI, a trauma-focused intervention is needed, whereas an intervention that excludes targeting MI as a trauma is not effective.

The primary aim of our randomized-controlled trial is to examine whether the development of posttraumatic stress can be reduced by a single counseling session of 45 minutes targeting MI-triggered traumatic reactions (that is, the *verum* intervention). The effect of this counseling session will be tested against a control intervention targeting the general role of psychosocial stress in CHD. The verum and control interventions are both administered to patients at high risk to develop PTSD within a setting of a coronary care unit (CCU) within 48 hours by the bedside after patients having reached stable circulatory conditions. Secondary aims of our trial are to investigate psychosocial functioning and cardiometabolic risk markers between the two active intervention arms.

Our main hypothesis is that trauma-focused psychological counseling is more effective than non-trauma focused counseling for reducing posttraumatic stress after acute MI. Specifically, we predict that compared with the control group, the verum group will report at least 20% lower posttraumatic stress levels at three months after MI as rated by blinded interviewers (primary outcome). We expect that this group difference will persist for up to 12 months. Compared to the control group, the verum group will additionally show better psychosocial functioning and a more favorable profile of cardiometabolic risk markers after 3 and 12 months of follow-up (secondary outcomes).

## Methods/Design

### Design

The study uses a randomized controlled two-armed design comparing a psychological counseling intervention (that is, the *verum* intervention) with an active control intervention after patient inclusion in the study. Sequence generation is done in a 1:1 fashion based on a computer-generated random list with randomly varying blocks of 20. The randomization sequence is generated by the website Research Randomizer (http://www.randomizer.org). The list is concealed by instructed office personnel in the study center of the Division of Psychosomatic Medicine, University of Bern, Switzerland, who is unaware of any patient information and localized off-site from the CCU. Study therapists receive the information to provide either the *verum* intervention or the control intervention directly from the study center.

### Sample size calculation

As opposed to the primary outcome, which is interviewer-rated posttraumatic stress, the sample size calculation is based on self-rated posttraumatic stress levels, which we had previously collected from almost 300 post-MI patients [12]. Based on these data we expect that patients at a high risk to develop PTSD (compare to below for definition) will show a mean score of 15.2 ± 10.1 (range 0 to 51) on the self-rated Posttraumatic Diagnostic Scale (PDS) at three months after MI. However, we cannot exclude a non-specific beneficial effect of the control intervention on PTSD symptom development. Therefore, we assume that patients in the control group will decrease their posttraumatic stress level by 1.0 PDS score point to 14.2 score points. We defined a 20% decrease in the posttraumatic stress level in the *verum* group compared to the control group to be clinically meaningful. Therefore, in order to achieve a 20% difference in posttraumatic stress levels between the *verum* intervention and the control group, the *verum* intervention should achieve a reduction of 3.9 PDS score points from 15.2 to 11.3 to show a clinically meaningful effect. Applying an alpha error level of *P* = 0.05, a beta error level of 20% and a group difference in the total PDS score of 2.9 ± 10.1, the sample size to detect a significant difference of 20% in posttraumatic stress levels between groups is n = 194 per group. Because of possible drop outs (including post-MI mortality), we will oversample each group by 10% (that is, n = 213 per group), yielding a total sample of n = 426 eligible post-MI patients to be randomly allocated to the two active intervention arms.

### Study sample

**Figure 1:**
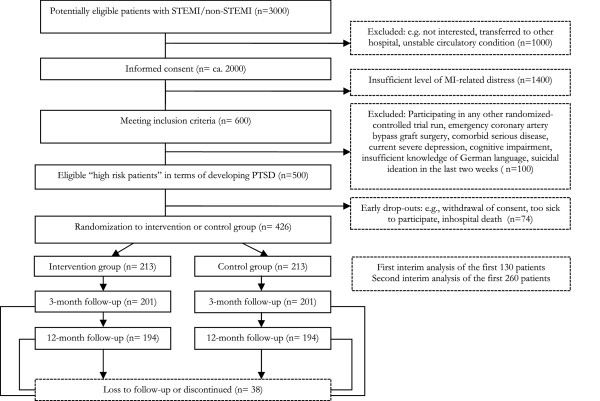
**Recruitment and participant flow.** MI, myocardial infarction; NSTEMI, non-ST-elevation myocardial infarction; STEMI, ST-elevation myocardial infarction; PTSD, posttraumatic stress disorder.

Based on the recruitment experience from previous studies on PTSD in post-MI patients and other trials of the study CCU, we predict to consecutively enroll the required number of 426 eligible patients for the study within 24 months (between 1 January 2013 and 31 December 2014). Figure 1 details the recruitment and participant flow. In brief, patients with ST-segment elevation MI (STEMI) and non-STEMI and referred to the CCU, Department of Cardiology, Inselspital, Bern University Hospital, Switzerland, are invited to participate in the study. The department’s cardiologists make a diagnosis of STEMI or non-STEMI per standard criteria as previously described [38]. The average biennial admission rate of patients with STEMI and non-STEMI to the CCU is approximately 3,000. Following a secondary analysis of a previous data set [12], about 900 (approximately 30%) of these will be 'high risk patients’ in terms of perceiving a substantial amount of distress during MI (compare to. paragraph below for precise definition). Of these, we predict that one third will not be accessible, mainly because they are transferred to other hospitals early after coronary interventions or will not provide consent, leaving 600 patients meeting inclusion criteria. One fourth of these will not be eligible because they meet exclusion criteria or drop out early.

MI-triggered distress to define 'high risk’ patients is assessed within 6 to 36 hours of hospital referral with three single-item questions asking about the intensity of pain, fear of dying and helplessness to be rated on a numeric scale ranging from 0 to 10. Very similar single-item questions referring to perceived distress during ACS have been used in previous studies showing good reliability and predictive value for psychosocial adaptation and cardiovascular prognosis [39,40]. Those scoring at least five points for 'pain during MI’ plus at least five points for 'fear of dying until admission to the CCU’ and/or 'worrying and feeling helpless when being told about having MI’ are 'high risk patients’.

### Timing of assessments

Patients will be assessed at three time points: on the CCU within 48 hours after MI (T0), at three months after discharge from the CCU (to assess acute posttraumatic stress) (T1), and at 12 months after discharge from the CCU (to assess chronic and late-onset posttraumatic stress) (T2). It is critical to approach patients at the earliest possible time after referral because they will usually stay for only 48 hours in the CCU before discharge. Therefore, within 12 to 24 hours of admission to the CCU (T0), all patients with STEMI/non-STEMI, aged 18 and older, and with stable circulatory condition will be informed about the planned study and are given a few hours to consider their participation. The study therapist will approach all eligible patients to ask about their willingness to participate in a single counseling session about the role of psychological stress in CHD and the possibilities to self-manage stress. Patients will be told that the provided information might be useful for good mental functioning while building up their life after MI. The patient will be told that if he/she agrees to participate he/she will be randomized to either counseling session A, targeting the more sudden and unexpected psychological stress which may occur in the aftermath of MI (that is, the *verum* intervention) or to counseling session B, targeting more general aspects about the role of psychosocial stress in MI (that is, the control intervention). During this information, study therapists avoid any terminology specifically pertaining to trauma. After having provided informed consent, and if reporting a sufficient amount of distress from MI, they will undergo medical and psychometric data assessment as specified below.

### Inclusion and exclusion criteria

Patients are included in the study if they are aged 18 or older, have STEMI or non-STEMI, are in a stable circulatory condition according to the treating cardiologist, and had perceived substantial distress during MI based on the three screening questions targeting pain, fear of dying, and helplessness. Patients are excluded from the study if they have undergone emergency coronary artery bypass graft surgery, suffer from a serious comorbid disease likely to cause death within one year, currently have current severe depression (per the cardiologist’s history and clinical impression), are not fully oriented and/or cognitively impaired, have insufficient knowledge of German, affirm suicidal ideation in the last two weeks, or are participating in another randomized controlled trial in the Cardiology Department.

### Intervention

Study therapists with a master’s degree in clinical psychology deliver the intervention within 48 hours of admission, as a single session of 45 minutes with the therapist by the bedside. Therapists are trained and supervised by senior clinical psychotherapists with degrees in psychology or psychiatry from the Clinical Psychology and Psychotherapy, Department of Psychology, University of Bern and Department of Psychiatry and Psychotherapy, University Hospital Zurich, Switzerland. Study therapists undergo at least 30 hours of training prior to beginning the trial. Two independent experts will rate 30 audiotaped intervention sequences to monitor adherence of study therapists to the intervention protocol. In addition, 50 patients from each intervention group will be asked to indicate their perception as to whether they belong to the intervention group A (that is, the *verum* group) or B (that is, the control group).

Table 1 details the components and topics of the two interventions. Each intervention consists of a counseling session of 45 minutes plus delivery of a colored information booklet for patients to deepen the oral information. Text and figures of the two booklets are specific to each mode of intervention and also serve as the basis for interacting with the patient. For the first half of the session, therapists will primarily cover topics that are of most immediate concern to the individual patient. For instance, when asking about previous stressful situations (in the control intervention) and the patient mentions a difficult situation at work, the discussion will be on job stress. Other forms of stressful life circumstances will not be covered but are dealt with in the information booklet. Nevertheless, during the course of counseling, therapists are required to inform about key topics, for instance, in the *verum* intervention, that it is a normal and common experience to perceive MI as stressful.

**Table 1:** Topics and questions covered by the two interventions

***Verum *group**	**Control group**
a) What is a trauma? Why can acute MI be understood as a traumatic experience?	a) What is psychosocial stress and when can it become dangerous?
b) Why can MI have a psychological impact?	b) Why do not all people react the same way to psychosocial stress?
c) How do patients cope with and adjust to their MI experience?	c) Which types of psychosocial stress are known to potentially have an influence on CHD and cardiac prognosis?
d) What are the most common reactions to traumatically experienced MI?	d) How can psychosocial stress affect a healthy life style, adherence to cardiac therapy, and cardiovascular biology?
e) Other reactions to traumatically experienced MI.	e) What can be done to reduce psychosocial stress?
f) What is PTSD, in general and related to MI?	
g) Why do not all patients react in the same way to MI?	
h) Coping with the trauma, tackling avoidance, coping with safety behaviors, anxiety, anger/irritability, sleeping problems, alcohol and medication	
i) How to get professional help	

CHD, coronary heart disease; MI, myocardial infarction; PTSD, posttraumatic stress disorder.

The 45-minute *verum* intervention uses an educational and resource-oriented approach targeting individual patient resources and cognitive (re)structuring to specifically prevent MI-triggered traumatic reactions. The study therapist will introduce the concept of psychological trauma and PTSD and how MI patients may deal and cope with posttraumatic stress symptoms should these occur after MI [36,37,41,42]. The focus of the 45-minute control intervention is to inform patients about the role of psychosocial stress in CHD in general and how this information might be considered in re-building a life after MI for the sake of proper mental functioning [43-47]. Most importantly, the control intervention will completely avoid any terminology specifically pertaining to 'trauma’, including the MI as a trauma.

### Measures

Table 2 shows all health outcomes in the MI-SPRINT study with the timing of their assessment. All psychometric questionnaires used in the MI-SPRINT study are validated in the German language [48-77]. We will apply the Clinician-Administered PTSD Scale (CAPS) to assess our primary outcome (that is, interviewer-rated posttraumatic stress level at three months after the intervention) [48]. Assessors will be blinded to the type of intervention and asked to indicate their guess as to whether the patient had received the *verum* intervention or the control intervention. The German version of the CAPS shows good internal consistency for the severity score of all 17 PTSD symptom items (Cronbach’s α = 0*.*88 to 0.92) as defined by the *DSMcpIV*[49]. The frequency and intensity of each symptom are rated between 0 (never) and 4 (almost always). A symptom is given when frequency is at least one point and intensity is at least two points. One of five symptoms is required for criterion B (reexperiencing cluster), three of seven symptoms for criterion C (avoidance cluster), and two of five symptoms for criterion D (hyperarousal cluster). The severity of PTSD is obtained by adding up symptom scores of criteria B+C+D. Patients will be diagnosed with syndromal PTSD if meeting criteria B+C+D [1].

**Table 2:** Measures at hospital referral (T0), three-month (T1) and twelve-month (T2) follow-up

**Variables**	**T0**	**T1**	**T2**
Sociodemographic and medical variables			
Demographics: for example, gender age, marital status	x		
Medical history: for example, family history of coronary artery disease, diabetes mellitus, hypertension	x		
Lifestyle and health behavior: smoking history, alcohol consumption, physical exercise, sleep quality	x	x	x
Prescribed medication before CCU referral	x		
Medication administered during CCU stay	x		
Medication at discharge from CCU	x		
Context of hospital referral: for example, patient agitation state at arrival on CCU, hectic conditions on CCU at patient arrival	x		
Clinical presentation at time of arrival at CCU: for example, total number of stents implanted, number of diseased coronary vessels, left ventricular function, Killip classification, pain onset	x		
Psychometric data			
Clinician-administered PTSD Scale (CAPS) [48,49]		x	x
Posttraumatic Diagnostic Scale (PDS) [50,51]	x	x	x
PTSD screening with three items from the structured clinical interview for DSM-PTSD [52,53]	x		
Acute Stress Disorder Scale (ASDS) [54,55]	x		
Illness Perception Questionnaire-Revised (IPQ-R) [56,57]	x		
Beck Depression Inventory (BDI) [58-60]		x	x
Type D Scale (DS14) [61,62]		x	
Toronto Alexithymia Scale (TAS-20) [63,64]		x	
Resilience Scale [65,66]	x	x	
EuroQol group five dimension questionnaire (EQ-5D) [67,68]	x	x	x
Symptom Checklist-9 (SCL-9-K) [69,70]	x	x	x
Enhancing Recovery in Coronary Heart Disease Social Support Inventory (ESSI) [71,72]	x	x	
Global Mood Scale (GMS) [73]		x	x
Coping Inventory for Stressful Situations (CISS) [74,75]		x	
Heart drawing [76,77]	x		
Laboratory Analysis			
Metabolic factors: total cholesterol, LDL-C, HDL-C, triglycerides, glucose, HbA1c Routine laboratory measures: alanine transaminase, creatinine, hemoglobin, hematocrit, leukocytes, thrombocytes	x	x	x
Inflammation markers: hs-CRP, IL-6, TNF-α, IL-4	x	x	x
Hemostasis markers: fibrinogen, D-dimer, VWF antigen	x	x	x
Stress hormones: plasma cortisol, norepinephrine, epinephrine	x	x	x
Heart rate variability: total power, high frequency power, low frequency power, low-to-high frequency power ratio	x	x	x
Resting hemodynamics: heart rate, systolic/diastolic blood pressure	x	x	x
Anthropometric measurements: body weight, height, body mass index; waist and hip circumferences, waist-to-hip ratio	x	x	x
Developments since discharge from CCU/three-month assessment			
Vitality status		x	x
Hospital referrals since discharge (including recurrent cardiovascular events)		x	x
General practioner visits		x	x
Specialist visits		x	x
Cardiac rehabilitation		x	x
Psychotherapy since discharge		x	x
Recurrent cardiac symptoms		x	x
Medication adherence		x	x
Functional status		x	x
Treatment adherence with information booklet		x	x
Major life events		x	x
Onset of new diagnosis		x	x
Current medication		x	x

CCU, coronary care unit; HbA1c, hemoglobin A1c; HDL-C, high-density lipoprotein-cholesterol; hs-CRP, high-sensitive C-reactive protein; IL-6, interleukin-6; IL-4, interleukin-4; LDL-C, low-density lipoprotein-cholesterol; TNF, tumor necrosis factor; VWF, von Willebrand factor.

### Cardiometabolic assessments

Fasting blood samples will be obtained at 6 am, within 24 hours after hospital referral, and directly sent to accredited laboratories at the Bern University Hospital to be processed within clinical routine on the same day. For catecholamine and cytokine analyses, ethylene-diamine-tetraacetic acid (EDTA) samples are centrifuged once at 2,000 g for ten minutes at room temperature; obtained plasma is frozen in CryoPure Tubes (Sarstedt, Numbrecht, Germany) at -80°C until analysis.

A Modular P800 system with ion selective electrodes and Cobas standard test kits (all Roche Diagnostics, Rotkreuz, Switzerland) will be used to determine levels of blood lipids, glucose, alanine transaminase, and creatinine from heparin plasma samples. Hemoglobin A1c will be determined in whole blood using high-performance liquid chromatography on a HLC-723 G7 analyzer (Tosoh Corp., Tokyo, Japan). Hemoglobin, hematocrit, leukocyte and thrombocyte counts will be determined from EDTA plasma samples using the LH 750 analyzer (Beckman Coulter, Miami, FL, USA).

Heparin plasma samples will be analyzed for high-sensitivity C-reactive protein by a latex particle immunoturbidimetric assay on the Roche Modular P800 analyzer (Roche Diagnostics, Rotkreuz, Switzerland). Levels of tumor necrosis factor-α, interleukin-6, and interleukin-4 will be measured in EDTA plasma samples using luminex technology (Bio-Plex Pro™ Cytokine Assays, Bio-Rad Laboratories AG, Cressier, Switzerland).

Hemostasis markers will be determined from citrate plasma samples. Fibrinogen clotting activity will be measured by the Clauss method on a Behring Coagulation system automated analyzer using commercial reagents and standard human plasma (Dade Behring AG, Düdingen, Switzerland). D-dimer levels will be determined through an enzyme-linked immunosorbent assay (Vidas-D-Dimer, bioMérieux, Geneva, Switzerland). A quantitative, automated immunoassay will be used to determine von Willebrand factor antigen (Siemens, Marburg, Germany).

Serum cortisol will be measured by competitive immunoassay using Roche Modular E170 (Roche Diagnostics, Rotkreuz, Switzerland). Norepinephrine and epinephrine concentration will be determined from EDTA plasma samples by means of HPLC and electrochemical detection after liquid-liquid extraction [78].

Heart rate and frequency domain measures of heart rate variability will be assessed from five-minute electrocardiogram recordings with the Finometer using customized software (TPD Biomedical Instruments, Amsterdam, The Netherlands) and averaged across the recording period. Blood pressure will be measured twice with an interval of ten minutes with the automatic Omron M6W Blood Pressure Monitor (Omron Healthcare Europe BV. Hoofddorp, The Netherlands); the average systolic and diastolic blood pressure will be computed for analysis.

The hip circumference measurement will be taken around the widest portion of the buttocks. The waist circumference will be measured at the approximate midpoint between the lower margin of the last palpable rib and the top of the iliac crest. The measurement of body weight will be performed with participants wearing no shoes and light clothing to the nearest 0.1 kg with a digital balance (Modell W41, Firma Migros, Switzerland) at follow-up assessments, and to the nearest 0.5 kg by history at hospital referral, because of potential fluid overload. Height will be measured with a reference tape to the nearest 0.1 cm. Body mass index will be calculated by dividing the weight in kilograms by height in meters squared.

### Ethical considerations

The study complies with the declaration of Helsinki. The ethics committee of the State of Bern, Switzerland, approved the study protocol. All potentially eligible patients (that is, those meeting the inclusion criteria) receive written information about the aim of the study, benefits and risks of participation and the exact study procedure before giving their written informed consent to participate in the study that includes two follow-up assessments. All participants are informed that they can withdraw themselves from the study anytime. All patients are encouraged to first contact their primary care physician in case they feel a need for mental health treatment at any time during follow-up. However, these patients will remain in the study, as it would be highly unethical to ask them to refrain from seeking professional help during follow-up. Patients who are diagnosed with syndromal PTSD at follow-up investigations are told their diagnosis. Moreover, patients and their primary care physicians are informed about the possibility of a referral to the Department of Clinical Psychology and Psychotherapy, University of Bern, Switzerland to receive treatment for PTSD.

To identify adverse reactions to the *verum* intervention, two interim safety analyses will be performed after subgroups of 130 and 260 patients on the primary efficacy endpoint, that is, interviewer-rated posttraumatic stress level at three months after the intervention. The spending function approximating O’Brien-Fleming boundaries [79] will be used to determine criteria for the interim and final analyses to protect the overall false positive rate of the trial at *P* = 0.05. The study will be stopped for inequality in outcome between the *verum* and control group if results reach a predefined level of statistical significance at the two-sided alpha level of 0.001 and 0.014, respectively. The nominal two-sided significance level for the final evaluation will be 0.045. This provides us with a 3.3% and 44.2% chance of stopping the trial after the first or second interim analysis, respectively.

### Statistical analyses

Data will be analyzed using the latest version of the Statistical Package for the Social Sciences (SPSS Inc. Chicago, IL, USA) with a significance level of *P* < 0.05 (two-tailed). Before analysis, all outcome variables will be tested for normality by the Kolmogorov-Smirnov test and, if necessary, transformed to obtain a normal distribution. Differences in variables between the two intervention groups will be calculated using the *t*-test for continuous data and the chi-squared test for categorical data. The primary outcome is the severity of posttraumatic stress levels rated by the CAPS interview at the three-month follow-up. Secondary outcomes are measures of psychosocial functioning and cardiometabolic biomarkers at three months follow-up (primary analysis). Key secondary outcomes are quality of life, plasma levels of C-reactive protein, D-dimer, cortisol and norepinephrine, as well as high-frequency heart rate variability. These outcomes are important indicators of mental health functioning, low-grade inflammation and coagulation activity as well as hypothalamic-pituitary adrenal axis and autonomic nervous system function. Repeated measures analysis of variance will be applied to test for a change in posttraumatic stress levels and in measures of psychosocial functioning and cardiometabolic biomarkers between the two follow-up visits (secondary analysis). The relative risk for developing a diagnosis of PTSD during follow-up in the control group compared to the *verum* group will be calculated by means of logistic regression analysis. For a complementary sensitivity analysis, we will apply univariate, multivariate and repeated measures analysis of covariance taking into account sociodemographic, medical and psychosocial covariates, and received psychotherapy during follow-up.

Missing values for questionnaire items will be replaced using the expectation maximization algorithm [80]. If patients drop out during follow-up, we will carry forward the last observed data points to impute missing endpoints. Randomized patients who will not undergo the intervention are excluded from the analysis. However, we will perform an additional analysis based on the intention-to-treat principle with all randomized patients.

## Discussion

The MI-SPRINT study is a randomized-controlled trial to investigate whether a minimal psychological intervention applied shortly after hospital referral may reduce the probability of developing clinician-rated posttraumatic stress levels attributable to MI. If effective, the planned trial will be the first to show that the development of posttraumatic stress can successfully be reduced in MI patients at high risk to develop PTSD through a single 45-minute counseling session that is feasibly (for example, not excessively time-consuming) administered in a busy CCU setting.

Cardiac patients with high risk to develop PTSD have generally limited access to early psychological care. Therefore it is important to know whether such an intervention bears the potential to become an integrative part of the post-acute treatment phase of patients with acute MI to improve quality of life and perhaps cardiovascular prognosis as well. Regarding the latter, a relative improvement of the atherothrombotic risk profile in the *verum* group might stimulate a larger intervention trial to possibly improve cardiac prognosis in patients with high distress after MI, as biologically plausible atherothrombotic mechanisms would seem to be responsive to an early psychological intervention.

There are limitations to the study design. Firstly, only those patients who are willing to participate in the study and to fill in questionnaires will be screened; therefore, it is possible that some severely distressed patients escape the screening, as they may feel too overwhelmed by the actual events for participating. Secondly, for feasibility reasons, we did not include non-German speaking patients, who constitute a significant part of the Swiss population. Therefore, the generalizability of our study findings to non-German speaking residents might be questioned. Nonetheless, the prevalence rates of PTSD in ACS patients do not vary greatly by study location (United States versus elsewhere, including Europe) [7]. Thirdly, although the control intervention avoids trauma terminology, an indirectly beneficial effect cannot fully be excluded, for instance if patients in the control group learn to better cope with general psychological distress. In this case, however, a group difference in posttraumatic stress levels that remains significant would only substantiate an assumed benefit of the trauma-focused *verum* intervention.

Several considerations on the design of the MI-SPRINT trial will need to be taken into account for the interpretation of study findings. We will assess posttraumatic stress at three-month follow-up, although ACS-induced PTSD symptoms at one-month might show greater variability and have been associated with recurrent CVD events [22]. Although we plan an exploratory analysis to investigate whether the intervention might offset recurrent CVD events, larger trials with adequate power would be needed for a reliable evaluation of this outcome. For both ethical and methodological reasons, we excluded patients with probably severe clinical depression who should receive standard antidepressant treatment, an imperative which would greatly complicate the protocol. A formal assessment of severe depression with a psychiatric interview is not feasible in this acute setting. A usual care control group might yield greater effects for the *verum* intervention, but such a design could not refute an interpretation that the mere contact with the study therapist was effective. We assess the subjective perception of the environment of acute medical care but for logistic reasons are unable to collect objective data on crowding or acuity of adjacent patients during participants’ CCU stay.

The MI-SPRINT study has several strengths; foremost its external validity, since MI patients are consecutively recruited from the cardiology department of a tertiary center with a wide rural and urban catchment area. In addition, the assessed psychometric and biomedical outcome variables and covariates are both well-established and cutting-edge measures in behavioral cardiology studies. To this extent, they may provide us with important novel insight into the prevention of posttraumatic stress development after MI and its link with psychosocial functioning and cardiometabolic risk, while also allowing us to identify potential moderating and/or confounding factors of these relationships. Finally, the MI-SPRINT study might extend our knowledge about the benefits and limits of psychological treatments for MI patients with a potential to reduce the burden on the health care system.

### Trial status

Recruitment of participants is ongoing; it began in January 2013 and is expected to end in December 2014. The final follow-up investigation is scheduled for December 2015.

## Abbreviations

ACS: acute coronary syndrome; CAPS: Clinician-Administered PTSD Scale; CCU: coronary care unit; CHD: coronary heart disease; CVD: cardiovascular disease; DSM-IV: *Diagnostic and Statistical Manual of Mental Disorders,* fourth edition; EDTA: ethylene-diamine-tetraacetic acid; HPLC: high-performance liquid chromatography; MI: myocardial infarction; MI-SPRINT: Myocardial Infarction - Stress PRevention INTervention; PDS: Posttraumatic Diagnostic Scale; PTSD: posttraumatic stress disorder; STEMI: ST-segment Elevation Myocardial Infarction.

## Competing interests

CH receives royalties for the self-help guide serving as the basis of our *verum* intervention.

## Authors’ contributions

RvK, JPS, US, JB, and HZ contributed to the development of the study design. US, HZ, JB, RvK, RM and MP contributed to the development of the *verum* and control intervention. CH provided the conceptual framework for the *verum* intervention. RM and MP wrote the first draft of the manuscript. All authors critically revised and approved the final manuscript.

## References

[B1] American Psychiatric Association Diagnostic and Statistical Manual of Mental Disorders 1994 4 Washington DC: APA

[B2] Gurevich M Devins GM Rodin GM Stress response syndromes and cancer: conceptual and assessment issues Psychosomatics 2002 43 4 259 281 10.1176/appi.psy.43.4.259 12189252

[B3] Tedstone JE Tarrier N Posttraumatic stress disorder following medical illness and treatment Clin Psychol Rev 2003 23 3 409 448 10.1016/S0272-7358(03)00031-X 12729679

[B4] Spindler H Pedersen SS Posttraumatic stress disorder in the wake of heart disease: prevalence, risk factors, and future research directions Psychosom Med 2005 67 5 715 723 10.1097/01.psy.0000174995.96183.9b 16204429

[B5] Schelling G Post-traumatic stress disorder in somatic disease: lessons from critically ill patients Prog Brain Res 2008 167 229 237 18037018 10.1016/S0079-6123(07)67016-2

[B6] Edmondson D Cohen BE Posttraumatic stress disorder and cardiovascular disease Prog Cardiovasc Dis 2013 55 6 548 556 10.1016/j.pcad.2013.03.004 23621964 PMC3639489

[B7] Edmondson D Richardson S Falzon L Davidson KW Mills MA Neria Y Posttraumatic stress disorder prevalence and risk of recurrence in acute coronary syndrome patients: a meta-analytic review PLoS One 2012 7 6 e38915 10.1371/journal.pone.0038915 22745687 PMC3380054

[B8] Abbas CC Schmid JP Guler E Wiedemar L Begré S Saner H Schnyder U von Känel R Trajectory of posttraumatic stress disorder caused by myocardial infarction: a two-year follow-up study Int J Psychiatry Med 2009 39 4 359 376 10.2190/PM.39.4.b 20391858

[B9] Guler E Schmid JP Wiedemar L Saner H Schnyder U von Känel R Clinical diagnosis of posttraumatic stress disorder after myocardial infarction Clin Cardiol 2009 32 3 125 129 10.1002/clc.20384 19301284 PMC6653086

[B10] Von Känel R Gander ML Hjemdahl P, Rosengren A, Steptoe A Posttraumatic Stress Disorder: Emerging Risk Factor and Mechanisms Stress and Cardiovascular Disease 2012 London U.K: Springer 235 256

[B11] Wiedemar L Schmid JP Müller J Wittmann L Schnyder U Saner H von Känel R Prevalence and predictors of posttraumatic stress disorder in patients with acute myocardial infarction Heart Lung 2008 37 2 113 121 10.1016/j.hrtlng.2007.03.005 18371504

[B12] Hari R Begré S Schmid JP Saner H Gander ML von Känel R Change over time in posttraumatic stress caused by myocardial infarction and predicting variables J Psychosom Res 2010 69 2 143 150 10.1016/j.jpsychores.2010.04.011 20624512

[B13] Edmondson D Shimbo D Ye S Wyer P Davidson KW The association of emergency department crowding during treatment for acute coronary syndrome with subsequent posttraumatic stress disorder symptoms JAMA Intern Med 2013 173 6 472 474 10.1001/jamainternmed.2013.2536 23400256 PMC3973030

[B14] Zatzick DF Marmar CR Weiss DS Browner WS Metzler TJ Golding JM Stewart A Schlenger WE Wells KB Posttraumatic stress disorder and functioning and quality of life outcomes in a nationally representative sample of male Vietnam veterans Am J Psychiatry 1997 154 12 1690 1695 9396947 10.1176/ajp.154.12.1690

[B15] McCrone P Knapp M Cawkill P Posttraumatic stress disorder (PTSD) in the Armed Forces: health economic considerations J Trauma Stress 2003 16 5 519 522 10.1023/A:1025722930935 14584638

[B16] Cohen BE Marmar CR Neylan TC Schiller NB Ali S Whooley MA Posttraumatic stress disorder and health-related quality of life in patients with coronary heart disease: findings from the heart and soul study Arch Gen Psychiatry 2009 66 11 1214 1220 10.1001/archgenpsychiatry.2009.149 19884609 PMC2822711

[B17] Boscarino JA A prospective study of PTSD and early-age heart disease mortality among Vietnam veterans: implications for surveillance and prevention Psychosom Med 2008 70 6 668 676 10.1097/PSY.0b013e31817bccaf 18596248 PMC3552245

[B18] Kubzansky LD Koenen KC Spiro A Vokonas PS Sparrow D Prospective study of posttraumatic stress disorder symptoms and coronary heart disease in the Normative Aging Study Arch Gen Psychiatry 2007 64 109 116 10.1001/archpsyc.64.1.109 17199060

[B19] Kubzansky LD Koenen KC Jones C Eaton WW A prospective study of posttraumatic stress disorder symptoms and coronary heart disease in women Health Psychol 2009 28 1 125 130 19210026 10.1037/0278-6133.28.1.125 PMC2757327

[B20] Scherrer JF Chrusciel T Zeringue A Garfield LD Hauptman PJ Lustman PJ Freedland KE Carney RM Bucholz KK Owen R True WR Anxiety disorders increase risk for incident myocardial infarction in depressed and nondepressed Veterans Administration patients Am Heart J 2010 159 5 772 779 10.1016/j.ahj.2010.02.033 20435185

[B21] Ladwig KH Baumert J Marten-Mittag B Kolb C Zrenner B Schmitt C Posttraumatic stress symptoms and predicted mortality in patients with implantable cardioverter-defibrillators: results from the prospective living with an implanted cardioverter-defibrillator study Arch Gen Psychiatry 2008 65 11 1324 1330 10.1001/archpsyc.65.11.1324 18981344

[B22] Edmondson D Rieckmann N Shaffer JA Schwartz JE Burg MM Davidson KW Clemow L Shimbo D Kronish IM Posttraumatic stress due to an acute coronary syndrome increases risk of 42-month major adverse cardiac events and all-cause mortality J Psychiatr Res 2011 45 12 1621 1626 10.1016/j.jpsychires.2011.07.004 21807378 PMC3210372

[B23] von Känel R Hari R Schmid JP Wiedemar L Guler E Barth J Saner H Schnyder U Begré S Non-fatal cardiovascular outcome in patients with posttraumatic stress symptoms caused by myocardial infarction J Cardiol 2011 58 1 61 68 10.1016/j.jjcc.2011.02.007 21493042

[B24] Zen AL Whooley MA Zhao S Cohen BE Post-traumatic stress disorder is associated with poor health behaviors: findings from the heart and soul study Health Psychol 2012 31 2 194 201 22023435 10.1037/a0025989 PMC3295904

[B25] Wentworth BA Stein MB Redwine LS Xue Y Taub PR Clopton P Nayak KR Maisel AS Post-traumatic stress disorder: a fast track to premature cardiovascular disease? Cardiol Rev 2013 21 1 16 22 10.1097/CRD.0b013e318265343b 22717656

[B26] von Känel R Hepp U Traber R Kraemer B Mica L Keel M Mausbach BT Schnyder U Measures of endothelial dysfunction in plasma of patients with posttraumatic stress disorder Psychiatry Res 2008 158 3 363 373 10.1016/j.psychres.2006.12.003 18252265

[B27] von Känel R Kraemer B Saner H Schmid JP Abbas CC Begré S Posttraumatic stress disorder and dyslipidemia: previous research and novel findings from patients with PTSD caused by myocardial infarction World J Biol Psychiatry 2010 11 2 141 147 10.3109/15622970903449846 20109110

[B28] Buckley TC Kaloupek DG A meta-analytic examination of basal cardiovascular activity in posttraumatic stress disorder Psychosom Med 2001 63 4 585 594 11485112 10.1097/00006842-200107000-00011

[B29] von Känel R Hepp U Kraemer B Traber R Keel M Mica L Schnyder U Evidence for low-grade systemic proinflammatory activity in patients with posttraumatic stress disorder J Psychotr Res 2007 41 9 744 752 10.1016/j.jpsychires.2006.06.009 16901505

[B30] von Känel R Begré S Abbas CC Saner H Gander ML Schmid JP Inflammatory biomarkers in patients with posttraumatic stress disorder caused by myocardial infarction and the role of depressive symptoms Neuroimmunomodulation 2010 17 1 39 46 10.1159/000243084 19816056

[B31] von Känel R Hepp U Buddeberg C Keel M Mica L Aschbacher K Schnyder U Altered blood coagulation in patients with posttraumatic stress disorder Psychosom Med 2006 68 4 598 604 10.1097/01.psy.0000221229.43272.9d 16868270

[B32] Roberts NP Kitchiner NJ Kenardy J Bisson J Multiple session early psychological interventions for the prevention of post-traumatic stress disorder Cochrane Database Syst Rev 2009 3 CD006869 10.1002/14651858.CD006869.pub2 19588408

[B33] Roberts NP Kitchiner NJ Kenardy J Bisson JI Systematic review and meta-analysis of multiple-session early interventions following traumatic events Am J Psychiatry 2009 166 3 293 301 10.1176/appi.ajp.2008.08040590 19188285

[B34] Rose S Bisson J Churchill R Wessely S Psychological debriefing for preventing posttraumatic stress disorder (PTSD) Cochrane Database Syst Rev 2002 2 CD000560 10.1002/14651858.CD000560 12076399

[B35] Ørner R Schnyder U Reconstructing Early Intervention After Trauma. Innovations in the Care of Survivors 2003 Oxford: Oxford University Press 36 38 Gestrichen (war text zu psychological debriefing)

[B36] Herbert C Understanding Your Reactions to Trauma. A Guide for Survivors of Trauma and Their Families. Revised Version 1995 Oxon: Blue Stallion Publications

[B37] Herbert C Wetmore A Overcoming Traumatic Stress. A Self-help Guide Using Cognitive Behavioral Techniques 2008 New York: Basic books

[B38] Kalesan B Stefanini GG Räber L Schmutz M Baumgartner S Hitz S Baldinger SH Pilgrim T Moschovitis A Wenaweser P Büllesfeld L Khattab AA Meier B Jüni P Windecker S Long-term comparison of everolimus- and sirolimus-eluting stents in patients with acute coronary syndromes JACC Cardiovasc Interv 2012 5 2 145 154 10.1016/j.jcin.2011.11.005 22361598

[B39] Whitehead DL Strike P Perkins-Porras L Steptoe A Frequency of distress and fear of dying during acute coronary syndromes and consequences for adaptation Am J Cardiol 2005 96 11 1512 1516 10.1016/j.amjcard.2005.07.070 16310432

[B40] von Känel R Hari R Schmid JP Saner H Begré S Distress related to myocardial infarction and cardiovascular outcome: a retrospective observational study BMC Psychiatry 2011 11 98 10.1186/1471-244X-11-98 21663602 PMC3126764

[B41] Reddemann L Dehner-Rau C Trauma. Folgen erkennen, überwinden und an ihnen wachsen. Ein Übungsbuch für Körper und Seele 2007 Stuttgart: Trias Verlag im Thieme Verlag

[B42] Ehlers A Clark DM Hackmann A McManus F Fennel M Herbert C Mayou R A randomized controlled trial of cognitive therapy, a self-help booklet, and repeated assessments as early interventions for posttraumatic stress disorder Arch Gen Psychiatry 2003 60 10 1024 1032 10.1001/archpsyc.60.10.1024 14557148

[B43] Rozanski A Blumenthal JA Davidson KW Saab PG Kubzansky L The epidemiology, pathophysiology, and management of psychosocial risk factors in cardiac practice: the emerging field of behavioral cardiology J Am Coll Cardiol 2005 45 5 637 651 10.1016/j.jacc.2004.12.005 15734605

[B44] von Känel R Psychological distress and cardiovascular risk. What are the links? J Am Coll Cardio 2008 52 25 2163 2165 10.1016/j.jacc.2008.09.015 19095134

[B45] Lazarus RS Folkman S Stress, Appraisal, and Coping 1984 New York: Springer Publishing Co

[B46] Dimsdale JE Psychological stress and cardiovascular disease J Am Coll Cardiol 2008 51 13 1237 1246 10.1016/j.jacc.2007.12.024 18371552 PMC2633295

[B47] von Känel R Psychosocial stress and cardiovascular risk: current opinion Swiss Med Wkly 2012 142 0 22271452 10.4414/smw.2012.13502

[B48] Blake DD Weathers FW Nagy LM Kaloupek DG Gusman FD Charney DS Keane TM The development of a Clinician-Administered PTSD Scale J Trauma Stress 1995 8 1 75 90 10.1002/jts.2490080106 7712061

[B49] Schnyder U Moergeli H German version of Clinician-Administered PTSD Scale J Trauma Stress 2002 15 6 487 492 10.1023/A:1020922023090 12482188

[B50] Foa EB Cashman L Jaycox L Perry K Validation of a self-report measure of posttraumatic stress disorder: the Posttraumatic Diagnostic Scale Psychol Assess 1997 9 4 445 451

[B51] Ehlers A Steil R Winter H Foa EB German Translation of the Posttraumatic Diagnostic Scale by Foa (1995) 1996 Oxford: Department of Psychiatry. Warneford Hospital

[B52] First MB Spitzer RL Gibbon M Williams JBW Structured Clinical Interview for Axis I DSM-IV Disorders 1995 New York: New York State Psychiatric Institute

[B53] Wittchen HU Zaudig M Fydrich T SKID-1. Strukturiertes Klinisches Interview für DSM-IV. Achse I: Psychische Störungen 1997 Göttingen: Hogrefe-Verlag

[B54] Bryant RA Moulds ML Guthrie RM Acute Stress Disorder Scale: a self-report measure of acute stress disorder Psychol Assess 2000 12 1 61 68 10752364

[B55] Helfricht S Landolt MA Moergeli H Hepp U Wegener D Schnyder U Psychometric evaluation and validation of the German version of the Acute Stress Disorder Scale across two distinct trauma populations J Trauma Stress 2009 22 5 476 480 10.1002/jts.20445 19760668

[B56] Galli U Ettlin DA Palla S Ehlert U Gaab J Do illness perceptions predict pain-related disability and mood in chronic orofacial pain patients? A six-month follow-up study Eur J Pain 2010 14 5 550 558 10.1016/j.ejpain.2009.08.011 19875320

[B57] Moss-Morris R Weinman J Petrie KJ Horne R Cameron LD Buick D The Revised Illness Perception Questionnaire (IPQ-R) Psychol Health 2002 17 1 1 16 10.1080/08870440290001494

[B58] Beck AT Steer RA Manual for the Beck Depression Inventory 1993 San Antonio TX: Psychological Corporation

[B59] Hautzinger M Bailer M Worall H Keller F Beck-Depressions-Inventar (BDI). Bearbeitung der deutschen Ausgabe. Testhandbuch 1994 Bern: Huber

[B60] Beck AT Ward CH Mendelson M Mock J Erbaugh J An inventory for measuring depression Arch Gen Psychiatry 1961 4 6 561 571 10.1001/archpsyc.1961.01710120031004 13688369

[B61] Denollet J DS14: standard assessment of negative affectivity, social inhibition, and Type D personality Psychosom Med 2005 67 1 89 97 10.1097/01.psy.0000149256.81953.49 15673629

[B62] Grande G Jordan J Kümmel M Struwe C Schubmann R Schulze F Unterberg C von Känel R Kudielka BM Fischer J Herrmann-Lingen C Evaluation der deutschen Typ-D-Skala (DS14) und Prävalenz der Typ-D-Persönlichkeit bei kardiologischen und psychosomatischen Patienten sowie Gesunden Psychother Psychosom Med Psychol 2004 54 413 422 10.1055/s-2004-828376 15494891

[B63] Parker JD Bagby RM Taylor GJ Endler NS Schmitz P Factorial validity of the Twenty-item Toronto Alexithymia Scale Eur J Personal 1993 7 221 232 10.1002/per.2410070403

[B64] Bach M Bach D de Zwaan M Serim M Validierung der deutschen Version der 20-item Toronto-Alexithymie-Skala bei Normalpersonen und psychiatrischen Patienten Psychother Psychosom Med Psychol 1996 46 1 23 28 8850096

[B65] Schumacher J Leppert K Gunzelmann T Strauss B Brähler E Die Resilienzskala - Ein Fragebogen zur Erfassung der psychischen Widerstandsfähigkeit als Personmerkmal Z Klin Psychol Psychiatr Psychother 2005 53 16 39

[B66] Wagnild GM Young HM Development and psychometric evaluation of the Resilience Scale J Nurs Meas 1993 1 2 165 178 7850498

[B67] Rabin R de Charro F EQ-5D: A measure of health status from the EuroQol Group Ann Intern Med 2001 33 5 337 343 10.3109/07853890109002087 11491192

[B68] Xie J Wu EQ Zheng ZJ Sullivan PW Zhan L Labarthe DR Patient-reported health status in coronary heart disease in the United States: age, sex, racial, and ethnic differences Circulation 2008 118 5 491 497 10.1161/CIRCULATIONAHA.107.752006 18625894

[B69] Derogatis LR SCL-90-R: Administration, Scoring, and Procedures Manual-I for the R(evised) Version 1993 Baltimore: John Hopkins University School of Medicine Baltimore

[B70] Klaghofer R Brähler E Konstruktion und teststatistische Prüfung einer Kurzform der SCL-90-R Z Klin Psychol Psychiatr Psychother 2001 49 2 115 124

[B71] Mitchell PH Powell L Blumenthal J Norten J Ironson G Pitula CR Froelicher ES Czajkowski S Youngblood M Huber M Berkman LF A short social support measure for patients recovering from myocardial infarction: the ENRICHD Social Support Inventory J Cardiopulm Rehabil 2003 23 6 398 403 10.1097/00008483-200311000-00001 14646785

[B72] Cámara RJ Lukas PS Begré S Pittet V von Känel R Effects of social support on the clinical course of Crohn’s disease Inflamm Bowel Dis 2011 17 6 1277 1286 10.1002/ibd.21481 21560191

[B73] Denollet J Emotional distress and fatigue in coronary disease: The global mood scale (GMS) Psychol Med 1993 23 1 111 121 10.1017/S0033291700038903 8475198

[B74] Kälin W Deutsche Kurzform des 'Coping Inventory of Stressful Situations’ (CISS) von NS Endler and JDA Parker. Basierend auf der Übersetzung von N Semmer et al. (unveröffentlichter Fragebogen) 1995 Bern: Universität, Institut für Psychologie

[B75] Endler NS Parker JD Multidimensional Assessment of coping: a critical evaluation J Pers Soc Psychol 1990 58 5 844 854 2348372 10.1037//0022-3514.58.5.844

[B76] Broadbent E Petrie KJ Ellis CJ Ying J Gamble G A picture of health–myocardial infarction patients’ drawings of their hearts and subsequent disability: a longitudinal study J Psychosom Res 2004 57 6 583 587 10.1016/j.jpsychores.2004.03.014 15596165

[B77] Rasband W Images [Free Software on the internet]. Version 1.46o USA: National Institutes of Mental Health [Cited 18 May 2012]. Available from: http://rsb.info.nih.gov/ij/

[B78] Ehrenreich H Schuck J Stender N Pilz J Gefeller O Schilling L Poser W Kaw S Endocrine and hemodynamic effects of stress versus systemic CRF in alcoholics during early and medium term abstinence Alcohol Clin Exp Res 1997 21 7 1285 1293 10.1111/j.1530-0277.1997.tb04450.x 9347091

[B79] Lan KK DeMets DL Discrete sequential boundaries for clinical trials Biometrika 1983 70 3 659 663

[B80] Enders CK A primer on the use of modern missing-data methods in psychosomatic medicine research Psychosom Med 2006 68 3 427 436 10.1097/01.psy.0000221275.75056.d8 16738075

